# Empirically Identifying and Computationally Modeling the Brain–Behavior Relationship for Human Scene Categorization

**DOI:** 10.1162/jocn_a_02043

**Published:** 2023-11-01

**Authors:** Agnessa Karapetian, Antoniya Boyanova, Muthukumar Pandaram, Klaus Obermayer, Tim C. Kietzmann, Radoslaw M. Cichy

**Affiliations:** Freie Universität Berlin, Germany; Charité – Universitätsmedizin Berlin, Einstein Center for Neurosciences Berlin, Germany; Bernstein Center for Computational Neuroscience Berlin, Germany; Technische Universität Berlin, Germany; Humboldt-Universität zu Berlin, Germany; Universität Osnabrück, Germany

## Abstract

Humans effortlessly make quick and accurate perceptual decisions about the nature of their immediate visual environment, such as the category of the scene they face. Previous research has revealed a rich set of cortical representations potentially underlying this feat. However, it remains unknown which of these representations are suitably formatted for decision-making. Here, we approached this question empirically and computationally, using neuroimaging and computational modeling. For the empirical part, we collected EEG data and RTs from human participants during a scene categorization task (natural vs. man-made). We then related EEG data to behavior to behavior using a multivariate extension of signal detection theory. We observed a correlation between neural data and behavior specifically between ∼100 msec and ∼200 msec after stimulus onset, suggesting that the neural scene representations in this time period are suitably formatted for decision-making. For the computational part, we evaluated a recurrent convolutional neural network (RCNN) as a model of brain and behavior. Unifying our previous observations in an image-computable model, the RCNN predicted well the neural representations, the behavioral scene categorization data, as well as the relationship between them. Our results identify and computationally characterize the neural and behavioral correlates of scene categorization in humans.

## INTRODUCTION

Humans effortlessly process visual input from their immediate environment to make adaptively relevant perceptual decisions (Henderson & Hollingworth, 1999). A large body of research has revealed a complex neural cascade involved in processing scenes, emerging across different brain regions (Grill-Spector, 2003; Hasson, Harel, Levy, & Malach, 2003; O'Craven & Kanwisher, 2000; Aguirre, Zarahn, & D'Esposito, 1998; Epstein & Kanwisher, 1998) and at different time points (Kaiser, Inciuraite, & Cichy, 2020; Cichy, Khosla, Pantazis, & Oliva, 2017; Harel, Groen, Kravitz, Deouell, & Baker, 2016). However, it remains unclear which representations are suited to guide behavior, as identification of activity related to a cognitive function does not imply that this activity can be translated into behavior. Instead, activity may, for example, be epiphenomenal (de-Wit, Alexander, Ekroll, & Wagemans, 2016), or be related to an interim processing stage that contributes to the creation of representations that later guide behavior but not do so themselves. To identify the representations that are suitably formatted to be used for decision-making, behavior and neural representations must be directly linked (Grootswagers, Cichy, & Carlson, 2018; Contini, Wardle, & Carlson, 2017).

**Figure 1.**
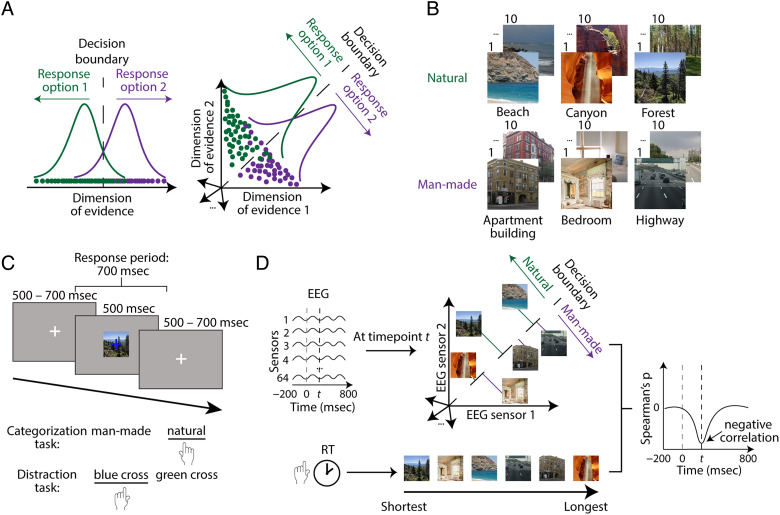
Stimulus set, paradigm, and distance-to-hyperplane approach. (A) Analysis approach. To link neural and behavioral data, we used the distance-to-hyperplane approach, an extension of signal detection theory in a multivariate space. (B) Stimulus set. We selected stimuli from Places-365 (Zhou et al., 2018), creating a set of 30 natural and 30 man-made scenes. (C) Experimental paradigm. Participants performed a scene categorization task on half of the blocks and an orthogonal fixation cross color detection task (referred to as distraction task) on the other half. (D) Distance-to-hyperplane approach. We applied the analysis at every time point to determine when neural representations are suitably formatted for decision-making, which occurs when the correlation between distances and RTs is significantly negative.

Here, we approached this challenge for scene perception from an empirical and a modeling perspective. For the empirical part, we obtained behavioral and EEG responses simultaneously from participants performing a scene categorization task. This ensured that the brain measurements directly corresponded to the observed behavior. We first applied time-resolved multivariate pattern analysis (Grootswagers, Wardle, & Carlson, 2017; Cichy, Pantazis, & Oliva, 2014) to reveal the time course with which scene representations emerge. To identify the subset suitably formatted for decision-making, we then related these unveiled scene representations to the RTs obtained in the scene categorization task. We did so by implementing a distance-to-hyperplane approach (Ritchie & Carlson, 2016; Figure 1A), a multivariate extension of signal detection theory (Green & Swets, 1966) previously used to link object representations measured with fMRI (Grootswagers et al., 2018; Carlson, Ritchie, Kriegeskorte, Durvasula, & Ma, 2014) and EEG (Contini, Goddard, & Wardle, 2021; Ritchie, Tovar, & Carlson, 2015) to behavior. Akin to the criterion in univariate space, it estimates a hyperplane in multivariate space that separates brain measurements for stimuli belonging to two different categories. The distance of the measurements for a stimulus to the hyperplane is assumed to determine behavior: Shorter RTs to the stimulus are associated with longer distances to the hyperplane and vice versa. Thus, in this framework, a negative correlation between RTs and distances to the hyperplane indicates that the investigated neural representations are suitably formatted for decision-making.

Subsequent to linking neural data and behavior, we used computational models to derive an image-computable model of scene categorization. We consider predictive models an integral part of understanding a scientific phenomenon (see Doerig et al., 2023; Lindsay, 2021; Saxe, Nelli, & Summerfield, 2021; Richards et al., 2019): If we understand a phenomenon, we should be able to provide a model of it that can itself be further evaluated by assessing the importance of model parameters linked to neural parameters (Cichy & Kaiser, 2019).

We formulate three desiderata for a suitable model of scene categorization: It should predict (1) the neural representations underlying scene categorization, (2) human scene categorization behavior, and (3) their relationship. A potential candidate class for the model are deep convolutional neural networks, which have been shown to predict activity in the visual cortex better than other models (Cichy et al., 2021; Schrimpf et al., 2020; Kietzmann et al., 2019; Yamins et al., 2014). A particular instantiation, a recurrent convolutional neural network (RCNN) named BLnet, that is, a model with learned bottom–up as well as lateral connectivity, has been shown to predict RTs in an object categorization task well and better than a range of control models (Spoerer, Kietzmann, Mehrer, Charest, & Kriegeskorte, 2020). Based on this observation, we evaluated BLnet, as well as a control, feedforward, parameter-matched network B-Dnet (Spoerer et al., 2020), with respect to their prediction of human visual scene representations, scene categorization RTs, and their relationship when relating the behavior of the model to human representations via the distance-to-hyperplane approach.

## METHODS

### Participants

Thirty healthy participants took part in the present study (mean age 22.7, *SD* = 2.82; 20 women, 10 men). We chose a sample size of 30 based on previous EEG studies using multivariate pattern analysis and the distance-to-hyperplane approach (Ritchie et al., 2015). All participants had normal or corrected-to-normal vision. All participants provided their informed consent after getting acquainted with the study protocol. The study was approved by the ethics committee of Freie Universität Berlin.

### Stimulus Set

The stimulus set was composed of 60 scene images selected from the validation set of Places-365 (Zhou, Lapedriza, Khosla, Oliva, & Torralba, 2018). They were center cropped and resized to 480 × 480. The set contained 30 natural and 30 man-made scenes (Figure 1B), each of the categories further divided into three categories of 10 stimuli (natural: beach, canyon, forest; man-made: apartment building, bedroom, highway).

### Experimental Design

Participants were presented with scenes in a random order, overlaid with a green or blue (randomly assigned) fixation cross, on a gray screen (Figure 1C). On each trial, the scene and fixation cross were presented for 500 msec and were followed by a jittered intertrial interval between 500 msec and 700 msec, where a gray screen and white fixation cross were shown.

Participants performed one of two separate tasks, categorization and fixation cross color identification (referred to as distraction; Figure 1C). They had 700 msec from stimulus onset to report their answer with a button press. In the categorization task, participants had to indicate whether a presented scene was natural or man-made. In the distraction task, participants had to report the color of the overlaid fixation cross. The key mapping was reversed on every block.

Categorization and distraction trials were presented in alternating blocks, of which there were 20 in total, 10 per task. One half of the participants started with one task, and the second half started with the other.

For the duration of the experiment, participants were asked to refrain from blinking during the scene trials and to only blink during trials on which a paperclip was shown, which for this purpose was presented for 1000 msec. Paperclip trials were regularly interspersed between main trials every three to five trials. Paperclip trials were not included in the analysis.

To ensure that enough data from correct trials (> 20 trials per scene) were available for each participant, every incorrect trial was repeated in the next block of the same task. Therefore, each block contained three trials per scene plus the scene trials that were misclassified in the previous block of the same task, as well as paperclip trials that constituted one fourth of all trials in a block. Because we only performed analyses on correct trials, this resulted in the inclusion of, on average, 23.2 (*SD* = 6.0) and 26.0 (*SD* = 1.46) trials per scene, respectively, for each task in the analyses.

Right before starting the data collection, participants performed two blocks of the paradigm containing 10 trials each to familiarize themselves with the paradigm.

The experiment was conducted in MATLAB (2019b) using Psychtoolbox (Brainard, 1997).

### EEG Recording and Preprocessing

Brain activity was recorded using EEG with the Easycap 64-electrode system and Brain Vision Recorder. The electrodes were arranged based on the 10–10 system. The participants wore actiCAP elastic caps, connected to 64 active scalp electrodes: 63 EEG electrodes and one reference (Fz). We sampled the activity with a 1000-Hz rate, which was amplified using actiCHamp and filtered online between 0.03 Hz and 100 Hz.

Offline, we preprocessed the EEG data using the FieldTrip toolbox (Oostenveld, Fries, Maris, & Schoffelen, 2010) in MATLAB (2021a, 2018b). First, we segmented the raw data into epochs of 1000 msec, using a prestimulus baseline window of 200 msec and a poststimulus window of 800 msec. Then, we performed baseline correction using the 200-msec prestimulus window. We applied a low-pass filter of 50 Hz, after which we downsampled the data to 200 Hz, resulting in 200 time points per epoch, each containing the average over 5 msec. To clean the data from artifacts, we used the automatic artifact rejection algorithm from the FieldTrip toolbox (Oostenveld et al., 2010). In addition, we manually removed noisy channels and trials (mean number of channels removed = 0.5, *SD* = 0.75, mean number of trials removed = 1, *SD* = 1.97). To control for the different levels of noise in the electrodes, we applied multivariate noise normalization (Guggenmos, Sterzer, & Cichy, 2018) by multiplying the data by the inverse of the square root of the covariance matrix of electrode activations from the entire epoch. The output of preprocessing was a time course of trial-wise patterns of electrode activations, which we used to perform the analyses described below.

### Scene Identity and Scene Category Decoding

We performed scene identity and scene category decoding on subject-level, trial-wise preprocessed EEG data from the categorization and distraction tasks by running classification analyses using a linear support vector machine (Vapnik, 1995) with the libsvm toolbox (https://www.csie.ntu.edu.tw/∼cjlin/libsvm/), in MATLAB (2021a). All four analyses contained three main steps, each performed independently on every time point. First, we transformed our data by averaging over individual trials to create “pseudotrials.” Second, we trained the classifier on a subset of data to predict either the scene identity or the scene category of given pseudotrials. Third, we collected the prediction accuracy of the classifier for the left-out data. After running the decoding analyses on all time points and participants, and averaging over participants, we obtained four time-courses, depicting scene identity and scene category decoding results for the categorization and distraction tasks.

First, we transformed the trial-wise preprocessed EEG data into pseudotrials by averaging over groups of trials of the same condition to boost the signal-to-noise ratio. To ensure that the training of the classifier was not biased, for each pairwise classification, we selected the same number of trials per scene in a random fashion, performing this selection and the rest of the analysis 100 times to make use of as much data as possible. For scene category decoding, this was followed by an extra step of splitting the trials from the natural and man-made categories into two groups, such that one half of all trials was used for training and the other half for testing. We then averaged over trials of the same condition (across four trials for scene identity and across 20 trials for scene category) to create pseudotrials. For scene category, this step was only performed for the training set: The testing set remained organized by scenes, because we were interested in scene-specific results for further distance-to-hyperplane analyses.

Second, we trained the classifier to predict stimulus conditions using a number of pseudotrials. For scene identity, we selected for the training set all but one pseudotrials. For scene category, half of all trials were selected to create the training pseudotrials, whereas the remaining half were used for the testing set. We trained the classifier to distinguish between patterns associated with different scenes in scene identity decoding (iterating over all pairwise combinations of scenes) or with the natural/man-made categories in scene category decoding.

Finally, we tested the classifier using the left-out data (the left-out pseudotrial for scene identity and the left-out half of all trials for scene category) to assess its prediction accuracy. We presented the classifier with data from two different conditions (depending on the analysis, either different scenes or scenes from different categories), to which it attributed condition labels, and recorded the accuracy of the prediction.

Performing this three-step analysis on all time points and all participants, and averaging over participants, resulted in four time-courses of the processing of neural representations associated with scene identity and scene category, in the categorization and distraction tasks.

### Distance-to-hyperplane Analysis

The distance-to-hyperplane analysis was performed using the following approach (Figure 1D). At every time point, we took the natural/man-made hyperplane estimated during category decoding and calculated, using the same left-out data, the distances to the hyperplane via decision values, which are a unitless measure provided as an output during support vector machine classification whose absolute value provides information about how close or far scene representations are from the hyperplane.

We then correlated the subject-level distances of all scenes with their RTs (median over participants) using the Spearman's rank-order correlation, at every time point, which resulted in one brain–behavior correlation time course per participant. After averaging over subject-level time courses, we identified the time points when the correlation was significantly negative, revealing when scene representations are suitably formatted to be used in decision-making.

### EEG Channel Searchlight Analysis

To identify the EEG channels whose signals indicated most the presence of representations associated with scene identity and scene category, as well as the ones that are suitably formatted for decision-making, we combined the decoding and distance-to-hyperplane approaches with a searchlight analysis in channel space. As we could not perform source reconstruction because of the lack of anatomical scans, we cannot identify where exactly the relevant brain activity is coming from. Instead, we make use of the information provided by the searchlight analysis to identify which channels are involved in the representations of interest, allowing us to approximately infer which regions of the brain contribute to the observed effect.

To implement the searchlight analysis, we used raw, nontransformed EEG data, just as for the whole-brain analysis, and performed the decoding and distance-to-hyperplane analyses separately on every channel, by taking into consideration the patterns over the channel and its four nearest neighbors. This resulted in topographic maps indicating for each channel the value of the decoding accuracy, in the decoding analyses, or of the correlation between distances to the hyperplane and RTs, in the distance-to-the hyperplane analysis.

### Fine-tuning and Feature Extraction of RCNN and Feedforward Convolutional Neural Network

To model scene categorization in humans, we selected a recurrent convolutional neural network (RCNN) BLnet (Spoerer et al., 2020) with lateral connections at every layer. In addition, to determine the role of recurrence in scene categorization modeling, we performed all analyses on a control network, the feedforward convolutional neural network (FCNN) B-Dnet (Spoerer et al., 2020). B-Dnet is the parameter-matched version of BLnet without the lateral connections. BLnet layers has in total seven layers, whereas B-Dnet has 14 layers.

Both networks were initially trained on ecoset (Mehrer, Spoerer, Jones, Kriegeskorte, & Kietzmann, 2021) for object classification, and we fine-tuned them on Places-365 (Zhou et al., 2018) in Tensorflow (Abadi et al., 2016) for scene categorization (natural vs. man-made). The training and validation sets for the fine-tuning consisted of samples from 80 scene categories (40 natural and 40 man-made), including the six categories from our stimulus set. The training set contained 30 samples per category, in total 2400 images, whereas the validation set contained 15 images per category, for a total of 1200 images. The images were center-cropped and resized to 128 × 128, and the pixel values were scaled to be in the range [−1, 1].

After fine-tuning, we fed the networks the 60 scenes that were used in the EEG experiment and collected features from three of their ReLU layers (early [Layer 1], mid-level [Layer 4 for RCNN, Layer 7 for FCNN], and late [Layer 7, for RCNN, Layer 14 for FCNN]), at each time step for RCNN (eight in total). We selected these layers to ensure that we sampled representations from throughout the hierarchy of the networks. This resulted in feature tensors that were further used in a representational similarity analysis (RSA) with EEG data to compare network and human scene representations.

### RSA between EEG and R/FCNN

We performed representational similarity analysis (RSA; Kriegeskorte, Mur, & Bandettini, 2008) in two steps: First, we constructed representational dissimilarity matrices (RDMs) for EEG and R/FCNN features, and afterwards, we correlated these RDMs.

#### Construction of RDMs

As a first step, we created RDMs, which are matrices containing dissimilarity values for different conditions, for EEG and R/FCNN separately.

To create the EEG RDMs, we used subject-level preprocessed EEG data to compute correlation distances (1-Pearson's coefficient) for each pair of scenes. To ensure that we are using an equal amount of data per condition, we first identified for each participant the minimum number of trials per scene and randomly selected that many trials for every scene. Then, we created pseudotrials by averaging over five trials for each scene. For each pair of scenes, we computed the correlation between a pair of pseudotrials. We performed this analysis for all pseudotrials and all pairwise combinations of scenes, 100 times with random assignment of trials to pseudotrials to select different subsets of trials every time. Averaging over all permutations and pseudotrials, we obtained one RDM per participant and time point. Because the RDMs are symmetric matrices, we only used the upper triangular matrix for the analysis (without the diagonal), which we vectorized in preparation for the next step.

To create the R/FCNN RDMs, we normalized the extracted features across scenes and calculated the correlation distance (1-Pearson's coefficient) between the features for two scenes for each pairwise combination of scenes, independently for every layer and RCNN time step. The upper triangular matrix of the RDM for each layer and RCNN time step was vectorized.

#### Correlation of EEG and R/FCNN RDMs

To compare the representations of humans and R/FCNN, we correlated (Spearman's coefficient) the subject-level EEG RDMs and R/FCNN RDMs. This correlation was performed independently for each participant, at each EEG time point and for each R/FCNN layer and RCNN time step, resulting in one correlation time course per layer, RCNN time step, and participant. After averaging over participants, we obtained a time course of similarity between scene representations of humans and R/FCNN for each of the three layers and each of the eight RCNN time steps. For RCNN, the final time courses depicted the median over the eight time steps. To compare the modeling results of RCNN and FCNN, we calculated and plotted their difference (BLnet − B-Dnet).

To determine whether CNNs and humans have different representational similarities depending on the supralevel category as previously observed for visual objects (Jozwik et al., 2022; Bracci, Ritchie, Kalfas, & Op de Beeck, 2019), we performed the analysis on all, natural, and man-made scenes separately, using the parts of the RDM for the respective scenes. For each of the three R/FCNN time courses, we collected the correlation peak latency, which represents the time point when EEG and network representations are most similar.

### Noise Ceiling

To determine the ideal correlation of EEG with R/FCNN given the noise levels in our data, we computed the noise ceiling (Nili et al., 2014). To calculate its lower bound, we correlated for each participant at each time point their EEG RDM with the average RDM over the rest of the participants. This resulted in one correlation time course per participant, and the final lower bound was obtained by averaging over all participants. The upper bound was calculated in a similar manner, except that the average RDM over participants also contained the RDM of the participant of interest.

### Collection of RCNN and FCNN RTs and Correlation with Human RTs

To model categorization behavior in humans, we extracted R/FCNN RTs according to the procedure described by Spoerer et al. (2020). The procedure was similar for RCNN and FCNN, but because FCNN does not have multiple readouts, we made slight adjustments. In general, according to the principles of threshold-based decision-making (Gold & Shadlen, 2007), humans make a decision (e.g., with a button press) once they accumulate enough evidence for a response option, at which point their RT is recorded. To collect RTs from a neural network in a comparable way, we can define a confidence level at which we say that the network has accumulated enough evidence to make a confident decision. Then, we calculate the number of time steps (or in the case of FCNN, the number of layers) required for the network to reach this confidence level, which represents the network's RT. The confidence level is defined here as a Shannon entropy threshold, which we select based on the fit with human data.

In detail, the procedure to collect RTs from the RCNN was as follows: (1) We defined an entropy threshold between 0.01 and 0.1; (2) we fed the 60 scenes used in the EEG experiment to the network in batches and collected the predictions (natural or man-made) from the readout layer, for each scene and each time step; (3) using these predictions, we calculated the Shannon entropy for each scene and each time step; (4) we collected the RTs, that is, for each scene, the first time step (between 1 and 8) that reached the entropy threshold. If the entropy was never reached, the RT was defined as 9.

The procedure was almost the same for FCNN, except instead of collecting predictions at every time step, we trained intermediate readouts after every second layer (seven in total) to predict the natural versus man-made scene category. Then, we performed the same entropy calculation as described above. The layer at which the entropy threshold was reached first constituted the RT. If the entropy threshold was never reached, the RT was defined as 8.

We repeated steps 1–4 of this procedure for 10 linearly spaced thresholds from 0.01 to 0.1. To select the final entropy threshold and the RTs associated with it, we correlated (Pearson's coefficient) network and human RTs for each of the 10 thresholds. This was performed in a cross-validated manner: For each fold (total 30, representing the number of participants), we left out the RTs from one participant (different participant at each fold) and correlated the median RTs over the remaining 29 participants with the network RTs. At each fold, the left-out participant's RTs were used to correlate (Pearson's coefficient) with network RTs for the final network-human RT correlation results. This correlation was performed for all scenes, natural and man-made, independently. For each fold, the selected entropy threshold and its associated RTs were the ones for which the network and median human RTs correlated the best. Here, the entropy threshold was the same for all folds (0.02 for RCNN and 0.06 for FCNN).

### Distance-to-hyperplane Analysis between EEG and R/FCNN RTs

Finally, to model the brain–behavior link, we performed the distance-to-hyperplane analysis between EEG data (distances to the hyperplane) and model RTs. We conducted this analysis with both RCNN and FCNN RTs. To compare the results for both models, we calculated and plotted the difference (BLnet – B-Dnet) in the EEG-RT correlation at every time point and every condition.

### Statistical Analysis

To assess the significance of our results, we performed nonparametric statistical tests.

We first created permutation samples using the sign-permutation test by randomly multiplying each participant's results ten thousand times either by 1 or −1. The *p* value of the original data was obtained by calculating the rank of its test statistic (mean divided by standard deviation) with respect to the distribution of the permutation samples.

Then, we controlled for multiple comparisons by adjusting the *p* values of the original data for their inflated false discovery rate (FDR) using the Benjamini–Hochberg procedure (Benjamini & Hochberg, 1995) with α = .01 for decoding and α = .05 for the rest of the analyses.

The test was right-tailed for all analyses except for the distance-to-hyperplane analysis (left-tailed) and the differences between RCNN and FCNN correlations with humans (two-tailed). We performed a left-tailed test on the distance-to-hyperplane results because our hypothesis only pertained to negative correlations (i.e., brain and behavior are correlated when shorter distances to the hyperplane are associated with longer RTs, and vice versa).

All peak latencies and their differences were tested for significance by bootstrapping the subject-level data 1000 times with replacement and calculating the 95% confidence intervals.

## RESULTS

### Behavior

The mean accuracy across participants for the categorization task was 79.2% (*SD* = 6.75) and 87.6% (*SD* = 5.89) for the distraction task.

The mean RT for the categorization task was 491.6 msec (*SD* = 35.9 msec) and 446 msec for the distraction task (*SD* = 36 msec).

### Scene Identity and Category Decoding Are Significant from ∼65 msec after Stimulus Onset until the End of the Trial

We began the neural investigation by revealing the time course with which scene representations emerge and develop over time in the brain using multivariate pattern analysis (also known as decoding; Cichy et al., 2014; Carlson, Tovar, Alink, & Kriegeskorte, 2013; Haynes & Rees, 2006; Kamitani & Tong, 2005). This had two aims: We first wanted to ensure that our measurements captured a rich set of candidate representations that could in principle be used by the brain for decision-making and, second, that our results were comparable to previous research, affording theoretical generalization of our results. For each participant and task context separately, we conducted two classification analyses: scene identity and scene category decoding for the natural versus man-made division, to reveal, respectively, the time course with which single images and scene categories were distinguishable by their neural representations.

**Figure 2.**
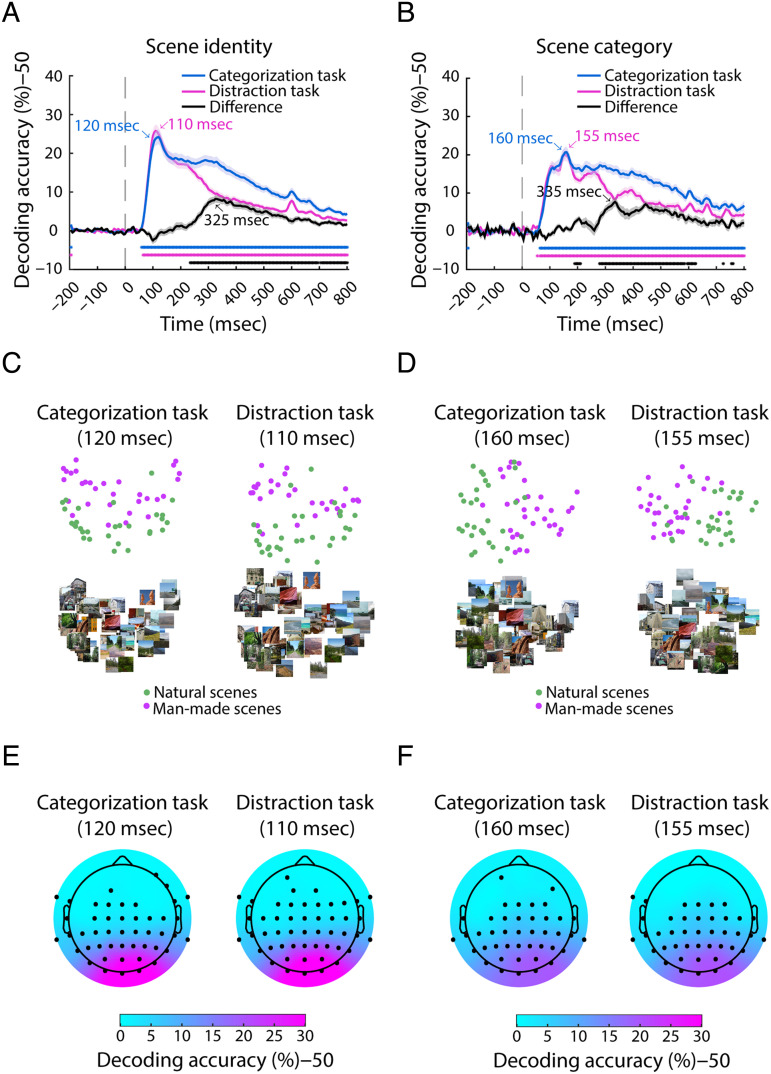
Scene identity and category decoding. (A) Pairwise scene identity decoding results on EEG data from the categorization task (blue), distraction task (magenta), and their difference (black). The vertical dashed gray line at 0 msec represents the stimulus onset. The shaded area around the curves indicates the *SEM*. Significant time points (right-tailed, *p* < .01, FDR-adjusted) are indicated with asterisks. (B) Scene category decoding (natural vs. man-made) results for both tasks and their difference. (C) Multidimensional scaling results for scene identity decoding from the categorization and distraction tasks at the scene identity decoding peak and (D) scene category decoding peak. (E) Results from the searchlight analysis performed in channel space in both tasks at peak decoding latency for scene identity decoding and (F) scene category decoding. Significant channels (right-tailed, *p* < .01, FDR-adjusted) are depicted with black dots.

We observed that for both scene identity and scene category decoding (Figure 2A and B, respectively), regardless of task, the representations of different conditions became consistently separable starting from ∼65 msec after stimulus onset (disregarding spurious effects before stimulus onset as expected statistically) and the conditions remained separable until the end of the trial (*p* < .01, FDR-adjusted for each analysis in this section). To visualize how the representations of scenes are distinguished in the brain, we performed multidimensional scaling on the results at peak scene identity (Figure 2C) and category (Figure 2D) decoding for each task. The plots show in each case separately—for both tasks and types of classification—that the representations of natural and man-made scenes at peak decodability are clearly separable.

Further inspection revealed a pattern of results concurrent with previous research in several key aspects. First, scene identity decoding peaked significantly earlier than natural versus man-made category decoding (∼115 msec vs. ∼160 msec, 95% confidence interval [CI] of the peak latency difference, averaged over tasks: [15, 52.5]), independently of task (for further details, see Table 1; Iamshchinina, Karapetian, Kaiser, & Cichy, 2022; Cichy et al., 2014; Carlson et al., 2013). Second, the time course for categorization and distraction tasks was similar during the early time points and at peak decoding but diverged around 200 msec, independent of the decoding scheme. Although this suggests that the task predominantly impacts feedforward processing of visual information (Hebart, Bankson, Harel, Baker, & Cichy, 2018; Harel, Kravitz, & Baker, 2014), it is likely that some recurrent processing has also taken place by then (Bullier, 2001). Third, a searchlight analysis in channel space (Figure 2E and F) revealed the topography of the electrodes carrying most information in the scene identity and category classification at the time of peak decoding. We observed a clear focus on occipital electrodes, suggesting that the neural sources of the relevant signals are likely, as expected, in the visual cortex (Graumann, Ciuffi, Dwivedi, Roig, & Cichy, 2022; Cichy et al., 2017).

**Table 1.** Statistical Details for Scene Identity and Scene Category Decoding

*Task*	*Type of Decoding*	*Peak Value*	*Peak Latency*	*95% CI* ^*a*^	*Significant Time Points* *
Categorization	Scene identity	74.2%	120 msec	[115, 120]	[−195, 60:800]
Distraction	Scene identity	75.8%	110 msec	[105, 115]	[−195, 65:800]
Difference	Scene identity	8.2%	325 msec	[315, 355]	[250:695, 705:800]
Categorization	Scene category	70.7%	160 msec	[145, 165]	[−195, 65:800]
Distraction	Scene category	70%	155 msec	[112.5, 165]	[55, 65:800]
Difference	Scene category	7.7%	335 msec	[325, 465]	[190:210, 280:585, 595:625, 725,755:760]

^a^ The confidence intervals were calculated by bootstrapping participants (*n* = 1000).

* Right-tailed, *p* < .01, FDR-adjusted.

Altogether, we verified using decoding that our data yield a temporal results pattern comparable to previous studies and capture a rich set of candidate representations potentially useful for decision-making. This forms a robust and experimentally well-anchored basis for our further investigation of the link between scene representations and behavior.

### Distances to the Hyperplane in Neural Space and Categorization RTs Are Negatively Correlated between ∼100 msec and ∼200 msec after Stimulus Onset

To determine when scene information encoded in neural representations is suitably formatted to be used for decision-making, we employed the distance-to-hyperplane approach (Ritchie & Carlson, 2016). This analysis consists of three steps, performed independently at every time point (Figure 1D). First, we estimated a natural/man-made hyperplane in neural space; second, we collected the distances of scenes to this hyperplane; and third, we correlated these distances with RTs to the same scenes (but different trials) from either a natural/man-made categorization task or the orthogonal distraction task. Applying the logic of signal detection theory (Green & Swets, 1966) to the neural space, we can identify the points in time at which scene representations and behavior are statistically linked. The rationale is that distance to a category criterion (here, the categorization hyperplane, estimated in a cross-validated way using a subset of trials) should predict categorization RT: Stimuli that are harder for humans to categorize should be associated with longer RTs and shorter distances to the hyperplane. Thus, a negative correlation between RTs and distances to the hyperplane in neural space links brain and behavior. However, we should not expect any such correlation with RTs from the distraction task because those RTs should not be guided by scene representations.

**Figure 3.**
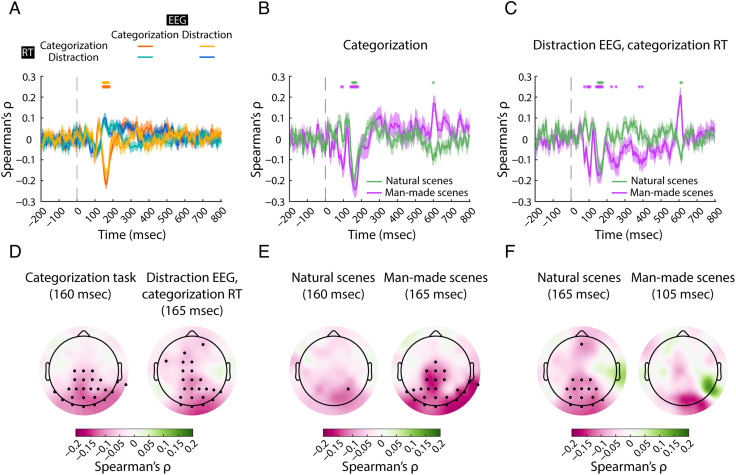
Distance-to-hyperplane analysis. (A) Results from the analysis on all 60 scenes, on data from the categorization task (orange), the distraction task (blue), using EEG from distraction and RTs from categorization (yellow), and EEG from categorization and RTs from distraction (turquoise). The vertical dashed gray line at 0 msec represents the stimulus onset. The shaded areas around the curves represent the *SEM*. Significant time points are denoted with asterisks (left-tailed, *p* < .05, FDR-adjusted). (B) Results from the within-categorization analysis on natural (green) and man-made (purple) scenes. (C) Results from the cross-task analysis using distraction EEG and categorization RTs. (D) Results from the searchlight analysis performed in channel space at peak negative correlation latency on all scenes in the categorization task (left) and cross-task using EEG data from distraction and RTs from categorization (right). (E) Results from the searchlight analysis on natural and man-made scenes in the categorization task and (F) the cross-task analysis with EEG from distraction and RTs from categorization. The negative correlations are in pink. Significant channels (left-tailed, *p* < .05, FDR-adjusted) are depicted with black dots.

We performed the distance-to-hyperplane analysis on data from each task separately, as well as across tasks (i.e., using EEG data from categorization and RTs from distraction, and vice versa), to determine the role of the task. We analyzed the data here and in subsequent analyses in three subsets: all scenes (Figure 3A), for a grand-average view; and natural and man-made scenes separately (Figure 3B and C), for a detailed category-resolved view. All three analyses relating EEG data and categorization behavioral data (Figure 3A, B, and C) converged in showing a significantly negative correlation (i.e., longer RTs were associated with shorter distances to the hyperplane, and vice versa) between ∼100 msec and ∼200 msec after stimulus onset, peaking at ∼160 msec with Spearman's ρ ≈ −.2 (left-tailed, *p* < .05, FDR-adjusted for each analysis in this section; see Table 2 for details). This demonstrates that neural representations arising during this time period are suitably formatted to act as basis for decision-making. Furthermore, it shows that these representations arise independently of whether the relevant categorization task or an unrelated task is carried out, indicating automaticity of the underlying processing (Harel et al., 2014).

**Table 2.** Statistical Details from the Distance-to-hyperplane Analysis

*Task*	*Condition*	*Peak Value*	*Peak Latency*	*95% CI* ^*a*^	*Significant Time Points* *
Within-task: categorization	All	ρ = −0.22	160 msec	[160, 165]	140–180
Within-task: categorization	Natural	ρ = −0.21	160 msec	[155, 165]	150–170, 600
Within-task: categorization	Man-made	ρ = −0.25	165 msec	[125, 175]	90–95, 140–180
Within-task: distraction	All	–	–	–	–
Within-task: distraction	Natural	–	–	–	–
Within-task: distraction	Man-made	–	–	–	–
Cross-task: categorization EEG, distraction RTs	All	*–*	–	–	–
Cross-task: categorization EEG, distraction RTs	Natural	ρ = −0.12	130 msec	[−77.5, 355]	125–130
Cross-task: categorization EEG, distraction RTs	Man-made	–	–	–	–
Cross-task: distraction EEG, categorization RTs	All	ρ = −0.17	165 msec	[150, 175]	145–175
Cross-task: distraction EEG, categorization RTs	Natural	ρ = −0.16	165 msec	[150, 615]	150–175, 610–615
Cross-task: distraction EEG, categorization RTs	Man-made	ρ = −0.18	105 msec	[100, 380]	75, 90–105, 140–175, 225, 250, 380, 395

^a^ The confidence intervals were calculated using bootstrapped permutation samples (*n* = 1000).

* Left-tailed, *p* < .05, FDR-adjusted.

For the control analyses relating EEG data with RTs from the scene category-unrelated distraction task (Figure 3A, blue and turquoise curves), there were no significant negative correlations between distances and RTs. This ascertains the specificity of the identified link between scene representations and classification behavior.

To identify the EEG channels whose signals indicated most the presence of representations that are suitably formatted for decision-making, we combined the distance-to-hyperplane approach with a searchlight analysis in channel space. In detail, we conducted one searchlight analysis (Figure 3D, E, and F) for each distance-to-hyperplane analysis described above (Figure 3A, B, and C) at the latency of the respectively identified peak. Consistent across all three analyses, we observed the strongest negative correlations in the occipital electrodes, suggesting the origin of the identified behaviorally relevant representations to be in the visual brain.

Interestingly, we observed that in certain analyses involving EEG from the distraction task (Figure 3D, right, and Figure 3F, left), a significant effect arose also in anterior electrodes overlying the frontal cortex. Although the difference between the results from the two tasks is not significant, this might still suggest that in contexts where automatic categorization is hindered by an unrelated task, frontal brain regions may contribute to representations of visual category suitably formatted for decision-making, consistent with the role of frontal cortex in processing object representations (Kar & DiCarlo, 2021; Bar et al., 2006; Bar, 2003; Freedman, Riesenhuber, Poggio, & Miller, 2001, 2003).

In summary, our results indicate that scene representations emerging automatically in the visual brain between ∼100 and ∼200 msec after stimulus onset are suitably formatted to be used for decision-making.

### RCNN Predicts Human Neural Representations, RTs, and the Brain–Behavior Relationship Better than FCNN

**Figure 4.**
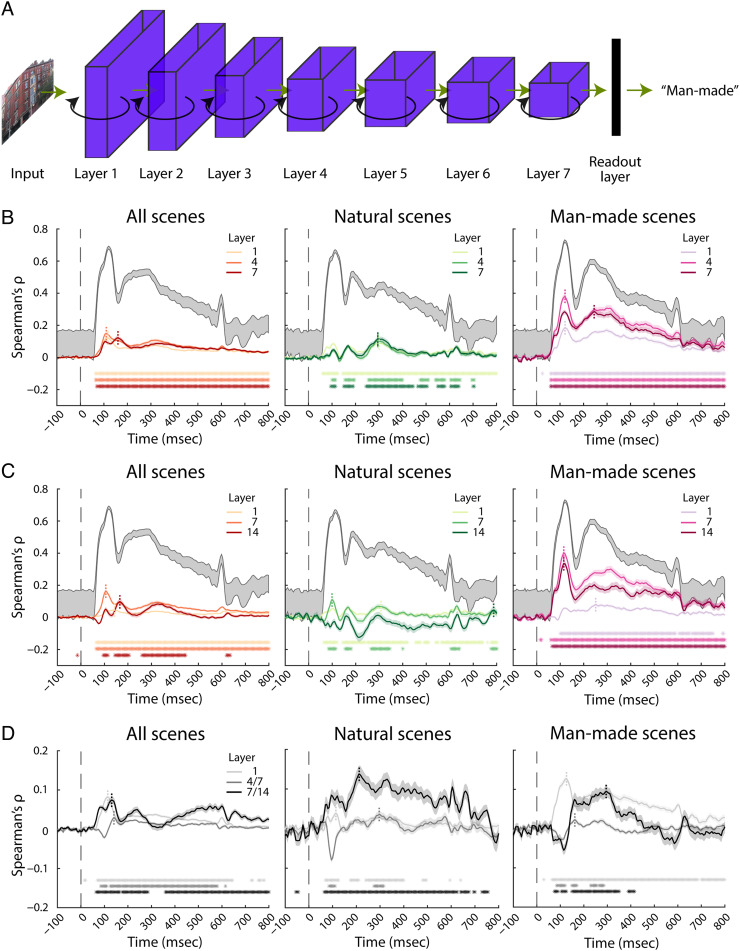
Modeling human neural scene representations with an RCNN versus an FCNN. (A) Architecture of BLnet (Spoerer et al., 2020), the recurrent CNN used in the analysis. The network consists of seven layers, linked via bottom–up (green arrows) and lateral (black arrows) connections. Features were extracted from three layers (1, 4, and 7) at eight different time steps, and RTs were collected from the readout layer. (B) Results of the RSA performed on the neural representations of humans and RCNN features from three different layers (median over eight time steps), for all scenes, natural scenes, and man-made scenes. The vertical dashed gray line at 0 msec represents the stimulus onset. The shaded areas around the curves represent the *SEM*. Significant time points are denoted with asterisks (right-tailed, *p* < .05, FDR-corrected). The dashed vertical lines indicate the peaks. The shaded gray area represents the noise ceiling. (C) RSA results with features from B-Dnet (Spoerer et al., 2020), the feedforward, parameter-matched version of BLnet. (D) Difference waves between RCNN and FCNN results (two-tailed, *p* < .05, FDR-corrected).

Based on the empirical work, we aimed at providing a computational model of scene categorization in humans. As a candidate model, we selected BLnet (Spoerer et al., 2020), an RCNN that has specifically been shown to predict RTs to objects, and that, as a deep neural network trained on an object classification task, belongs to the class of models that predict human visual cortex activity well (Cichy et al., 2021; Schrimpf et al., 2020; Kar, Kubilius, Schmidt, Issa, & DiCarlo, 2019; Kietzmann et al., 2019). The model consists of seven layers and contains bottom–up as well as lateral recurrent connections at every layer (see Figure 4A for architecture details). These lateral connections provide features from eight different time steps at each layer. The model was trained on object classification, and as training material specificity has been shown to impact predictive power for brain representations (Cichy et al., 2017; Mehrer et al., 2021; Cichy, Khosla, Pantazis, Torralba, & Oliva, 2016; Yamins et al., 2014), we first fine-tuned all its layers on a scene categorization task (natural vs. man-made) using a database of scenes (Zhou et al., 2018).

To determine whether recurrence improves the goodness of fit of CNN models in predicting human scene categorization, we also assessed the predicting power of a feedforward, parameter-controlled version of BLnet, namely, B-Dnet (Spoerer et al., 2020). This network has exclusively feedforward connections, but because of its seven additional layers, it contains the exact same number of parameters as BLnet, enabling us to directly evaluate the effect of recurrence on modeling of human data.

The fine-tuned recurrent network performed very well on the scene categorization task, reaching 98% accuracy on all scenes, 99% on natural scenes, and 97% on man-made scenes. However, the fine-tuned feedforward network performed worse, scoring 82% on all scenes, 100% on natural scenes, and 63% on man-made scenes.

We assessed three desiderata for a suitable model of scene categorization: The model should predict (1) the neural representations underlying scene processing, (2) human behavior, and (3) their relationship. For the first desideratum, we compared human and BL/B-Dnet representations using RSA (Kriegeskorte et al., 2008). For the model, we focused on three layers: early (Layer 1), mid-level (Layer 4 for BLnet, Layer 7 for B-Dnet), and late (Layer 7 for BLnet, Layer 14 for B-Dnet) as representatives for respective visual processing stages and performed the analysis on all eight time steps for BLnet. For the EEG, we built RDMs in a time-resolved fashion for every time point containing the average of 5 msec. Comparing RDMs (Spearman's coefficient) yielded time courses where positive correlations indicate similar scene representations in humans and BL/B-Dnet.

For BLnet, consistent across the analyses on all, natural and man-made scenes (Figure 4B), we observed, starting from ∼60 msec, significant positive correlations for all of the trial duration (right-tailed, *p* < .05, FDR-adjusted for each analysis in this section). Assessing the model's different time steps yielded comparable results; therefore, Figure 4B depicts the median over all time steps.

Focusing in detail on the analysis of all scenes (Figure 4B, left), we observed a forward shift in peak latency with increased network depth. In Layers 1 and 4, the correlation peaked at ∼110 msec, whereas in Layer 7, the peak correlation was significantly later, at 160 msec (95% CI of difference between Layers 1 and 4, and 1 and 7: 47.5 msec [20 65]; see Table 3 for further details), suggesting a temporal correspondence between network layer and processing stage at which different scene representations emerge (Greene & Hansen, 2018; Eickenberg, Gramfort, Varoquaux, & Thirion, 2017; Cichy et al., 2016; Güçlü & van Gerven, 2015; but see Sexton & Love, 2022). Focusing on the finer distinction between natural and man-made scenes (Figure 4B, middle and right), we observed overall weaker effects for natural scenes. Nevertheless, the results show that the model fulfils the first criterion of predicting human scene representations for all types of scenes.

**Table 3.** Statistical Details for the RSA between Humans and RCNN

*Layer*	*Condition*	*Peak Value*	*Peak Latency*	*95% CI* ^*a*^	*Significant Time Points* *
1	All	ρ = 0.12	115 msec	[110, 120]	65–800
1	Natural	ρ = 0.08	300 msec	[100, 320]	[60–125, 145–800]
1	Man-made	ρ = 0.19	120 msec	[120, 300]	[25, 65–800]
4	All	ρ = 0.15	110 msec	[105, 120]	60–800
4	Natural	ρ = 0.1	295 msec	[290, 315]	[100–115,155–195,255–400, 475–510, 540–580, 605–645, 695–705]
4	Man-made	ρ = 0.38	120 msec	[115, 120]	60–800
7	All	ρ = 0.12	160 msec	[130, 175]	70–800
7	Natural	ρ = 0.11	295 msec	[290, 325]	[95–115, 150–190, 245–445, 465–505, 550–580, 605–640, 700–70]
7	Man-made	ρ = 0.28	245 msec	[115, 315]	60–800

^a^ The confidence intervals were calculated using bootstrapped permutation samples (*n* = 1000).

* Right-tailed, *p* < .05, FDR-adjusted.

We observed weaker and more sparse correlations for the feedforward network, B-Dnet (Figure 4C), for all conditions. The difference waves (Figure 4D) demonstrate that RCNN correlates significantly better with EEG than FCNN for many time points throughout the time course. In particular, early and late layers benefit from recurrence, leading those layers in RCNN to better predict EEG data than the comparably deep layers in FCNN, across stimulus conditions. Overall, not only does the selected RCNN, BLnet, predict human scene representations well, but it predicts them better than its feedforward counterpart, B-Dnet.

To assess our second desideratum, that is, the prediction of behavior in terms of RTs in a categorization task, we compared RTs from BLnet/B-Dnet and humans. In short, we extracted network RTs (Spoerer et al., 2020) by fitting an entropy threshold in a cross-validated way and collecting the network time step (or layer) at which this entropy threshold was reached for each of the scenes, which then served as RT. We then correlated these network RTs with human RTs. As before, this analysis was performed separately for all, natural, and man-made scenes.

**Figure 5.**
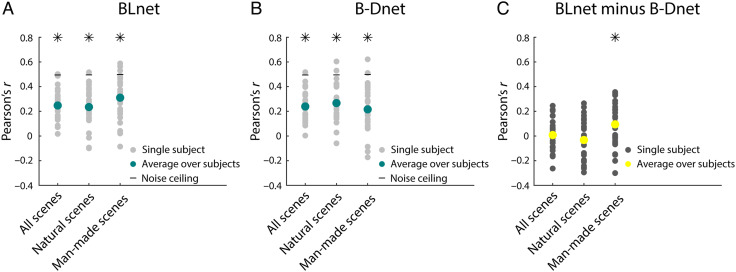
Modeling human RTs with an RCNN versus an FCNN. (A) Correlation between human and RCNN scene categorization RTs for all, natural and man-made scenes. Significant correlations are indicated with asterisks above the plot (right-tailed, *p* < .05, FDR-corrected). (B) Correlation between human and FCNN RTs. (C) Difference between RCNN and FCNN results (two-tailed, *p* < .05, FDR-corrected).

For BLnet (Figure 5A), for all three analyses, we observed significant positive correlations (Pearson's *r* = .25, .24, and .31, respectively; right-tailed, *p* < .05, FDR-adjusted), demonstrating that BLnet's RTs significantly correlate with human behavior.

B-Dnet also showed significant correlations with human RTs (Figure 5B). However, BLnet correlations were significantly higher for man-made scenes (two-tailed, *p* < .05, FDR-adjusted; Figure 5C), suggesting that recurrence improves the prediction of human scene categorization behavior for a subset of scenes.

Lastly, we assessed our third desideratum, that is, whether there is an analogy for BLnet/B-DNet and humans in the relationship between representations and behavior. For this, we conducted the distance-to-hyperplane analysis using human EEG data and network RTs. This analysis is nontrivial, as the correlation between human and network RTs is not so strong as to automatically suggest a significant correlation between network RTs and EEG.

**Figure 6.**
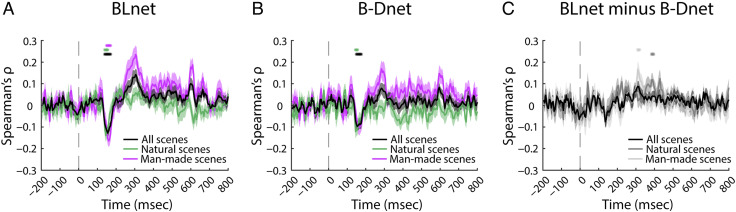
Modeling human brain–behavior link with an RCNN versus an FCNN. (A) Results of the distance-to-hyperplane analysis performed with distances from EEG data and RCNN RTs. The vertical dashed gray line at 0 msec represents the stimulus onset. The shaded areas around the curves represent the *SEM*. Significant time points are denoted with asterisks (left-tailed, *p* < .05, FDR-adjusted). (B) Results of the distance-to-hyperplane analysis performed with distances from EEG data and FCNN RTs. (C) Difference waves between RCNN and FCNN results (two-tailed, *p* < .05, FDR-adjusted).

Nevertheless, we observed that this analysis consistently yielded a significant negative correlation for BLnet RTs and EEG between ∼100 msec and ∼200 msec after stimulus onset (left-tailed, *p* < .05, FDR-adjusted; Figure 6A), analogous to the results from the analysis based on human rather than model RTs (see Figure 3A, B). This demonstrates that BLnet successfully mirrors the relationship between human visual scene representations and behavior.

Performing the analysis with B-Dnet RTs (Figure 6B) resulted in a significant negative correlation between ∼100 msec and ∼200 msec for all and natural scenes, but not for man-made scenes. This suggests that recurrence helps the prediction of the brain–behavior link for a subset of stimuli, man-made scenes.

In summary, we find that the recurrent model, BLnet, predicts well neural representations, RTs and the link between brain and behavior, significantly better than its feedforward counterpart B-Dnet for certain stimuli, thereby fulfilling all three formulated desiderata for a suitable computational model of visual scene categorization in humans.

## DISCUSSION

We investigated scene processing in humans using multivariate analyses of EEG data, RT measurements, and computational modeling based on an RCNN, BLnet (Spoerer et al., 2020). We highlight two main findings. First, using the distance-to-hyperplane approach on the empirical EEG and behavioral data, we found that neural representations of scenes are negatively correlated with RTs between ∼100 msec and ∼200 msec after stimulus onset, indicating that neural representations are then suitably formatted for decision-making. Second, we demonstrated that an RCNN is a good predictor of neural representations, behavior and the brain–behavior relationship for scene categorization, surpassing its feedforward counterpart and fulfilling all three desiderata that we formulated to identify a suitable model of scene categorization in humans.

### Neural Representations of Scenes Are Suitably Formatted for Decision-making between ∼100 msec and ∼200 msec after Stimulus Onset

Using brain decoding, we revealed that individual scenes and scene categories are represented in the brain continuously starting from ∼65 msec post stimulus, with peaks between 100 msec and 200 msec (Greene & Hansen, 2020; Kaiser et al., 2020; Cichy et al., 2017; Harel et al., 2016; Groen, Ghebreab, Prins, Lamme, & Scholte, 2013). Resolving the time course of scene representations does not, however, reveal conclusively when these representations are suited for use during decision-making. Performing the distance-to-hyperplane approach, we showed this to be the case in a short time-window between ∼100 msec and ∼200 msec post stimulus, coinciding with peak decoding.

The timing of the brain–behavior relationship is consistent with previous univariate studies (Greene & Hansen, 2020; Philiastides, Ratcliff, & Sajda, 2006; Philiastides & Sajda, 2006; VanRullen & Thorpe, 2001) and studies investigating other visual contents using other measurements of behavior, such as perceived similarity in abstract stimuli (Wardle, Kriegeskorte, Grootswagers, Khaligh-Razavi, & Carlson, 2016), objects (Cichy, Kriegeskorte, Jozwik, van den Bosch, & Charest, 2019; Bankson, Hebart, Groen, & Baker, 2018), and scenes (Greene & Hansen, 2020). This consistent temporal pattern for different visual contents suggests similar underlying neural mechanisms through which visual representations suitably formatted for behavior emerge.

We observed the brain–behavior relationship even for brain data recorded during a visual task unrelated to categorization behavior (Grootswagers et al., 2018; Ritchie et al., 2015; Carlson et al., 2014; Harel et al., 2014). It is consistent with our decoding results that show that scenes are represented in an automatic, task-independent manner in the early time points, coinciding with when the brain–behavior relationship emerges. Along with previous evidence that relevant scenes can be accurately categorized even when attention is focused on a different task (Li, VanRullen, Koch, & Perona, 2002), our results support the view of categorization as a core cognitive function of the visual system (DiCarlo, Zoccolan, & Rust, 2012; Grill-Spector, 2003; VanRullen & Thorpe, 2001). Moreover, the late (> 200 msec) dissociation of representations by task context, as also previously reported by Yip, Cheung, Ngan, Wong, and Wong (2022) and Farzmahdi, Fallah, Rajimehr, and Ebrahimpour (2021), shows that tasks may most strongly shape visual representations late in the processing hierarchy. For example, attention and task-related information arising in the frontal areas may update the representations in the lower-visual areas such that the visual cortex can favor and retain the information that is useful for behavior (Hebart et al., 2018). In summary, this finding highlights the significance and automaticity of categorizing perceptual information and of processing it into a behaviorally guiding format, enabling quick and adaptive decision-making.

Interestingly, we observed a relationship between brain measurements and behavior in frontal electrodes when relating RTs from scene categorization to EEG from the distraction task. Although we cannot infer the neural sources of the signal from the results of this analysis, they suggest that representations suited for behavior could be formed not only in the visual cortex, but also in the frontal regions. This finding would follow Grootswagers and colleagues (2018), who observed the activity in the prefrontal cortex during an orthogonal task, and is consistent with evidence from previous animal and human studies suggesting that frontal areas relevant for task performance are activated in decision-making (McGinty & Lupkin, 2021; Stringer, Michaelos, Tsyboulski, Lindo, & Pachitariu, 2021; Philiastides & Sajda, 2007; Heekeren, Marrett, Ruff, Bandettini, & Ungerleider, 2006; Kim & Shadlen, 1999; Hanes & Schall, 1996). Further research is required to specify the nature and cortical source of this effect.

### Recurrence Favors a Suitable and Unified Model of Human Scene Categorization

We showed that an RCNN, BLnet, was well suited for modeling human scene categorization on all levels and better than its feedforward match for certain stimuli. First, it performed the scene categorization task with a high accuracy and better than the parameter-matched FCNN. Furthermore, we observed a positive correlation between the representations of the recurrent network and humans from ∼60 msec poststimulus until the end of trial, consistent with previous findings of representational similarities between humans and CNNs in scene (Greene & Hansen, 2018; Cichy et al., 2017) and object (Kietzmann et al., 2019; Seeliger et al., 2018; Jozwik, Kriegeskorte, Storrs, & Mur, 2017; Cichy et al., 2016) recognition. Our results therefore add evidence toward the idea that CNNs, in particular, RCNNs are good predictors of human neural representations (Güçlü & van Gerven, 2015; Cadieu et al., 2014; Khaligh-Razavi & Kriegeskorte, 2014; Yamins et al., 2014), particularly for scenes (Doerig et al., 2022). Although the RCNN best predicted early EEG data (< 200 msec), it also predicted well later time points, suggesting that recurrent networks are good models of brain activity throughout the processing time course. In addition, we showed that RCNN representations correlated better with human representations than its feedforward, parameter-matched counterpart, reinforcing the specific importance of the role of recurrence in modeling the visual cortex (Kar et al., 2019; Kietzmann et al., 2019).

Having similar representations to humans is not enough for a network to qualify as a suitable model of human vision: It must also behave similarly to humans. This was the case for BLnet: We observed for the first time a positive correlation between human and network scene categorization RTs for all types of scenes. Our results contribute to efforts comparing human and CNN behavior in terms of performance (Seijdel, Tsakmakidis, Bohte, & Scholte, 2020), similarity judgments (King, Groen, Steel, Kravitz, & Baker, 2019; Jozwik et al., 2017), error consistency (Geirhos et al., 2021), and RTs (Rafiei & Rahnev, 2022; Sörensen, Bohté, de Jong, Slagter, & Scholte, 2022). In particular, we extend previous results relating RCNNs to human behavior from object recognition (Spoerer et al., 2020) to scene categorization, demonstrating the potential of RCNNs as models for diverse visual human behaviors. We also showed that the tested RCNN was better at predicting RTs for a subset of stimuli (man-made scenes) than its feedforward counterpart. This suggests that recurrence is useful for modeling scene categorization behavior.

Lastly, we observed that the relationship between EEG distances and network RTs between ∼100 msec and ∼200 msec post stimulus corresponded directly to the empirical results from the within-human analysis, suggesting that RCNNs can be used to successfully model the brain–behavior link. It could additionally imply that the representations that are similar in humans and RCNN are the ones feeding into scene categorization behavior. The feedforward network exhibited such a relationship with EEG for all and natural scenes, but not for man-made scenes, suggesting that recurrence benefits modeling the brain–behavior link in human scene categorization. This brings the analysis full circle by integrating brain measurements, behavior, and deep networks in a unified modeling account of human perceptual decision making.

However, the modeling results should be interpreted with caution: Although the tested RCNN correlated strongly with humans both in terms of neural representations and behavior, further research is needed to identify how much variance it explains in human data. There is room for improvement through better models, for example, by employing different objective functions, adding top–down connections, adding more parameters, and so on, to close the gap between computer and human vision (Zador et al., 2022; Geirhos et al., 2021; Kietzmann et al., 2019).

Furthermore, we observed an advantage of recurrence in terms of behavior and brain–behavior link only for man-made and not natural scenes. Given that in the within-human analysis the behavior-EEG correlation was also stronger for man-made than natural scenes (although we do not have statistical tests to support this), this is likely a signal-to-noise ratio issue. Further research focusing on natural images with increased signal-to-noise ratio is needed to resolve this issue.

### Limitations and Future Directions

There are several limitations to our results. First, the distance-to-hyperplane approach operationalizes behavior only in terms of RTs. It is possible that other neural representations are linked to other aspects of behavior, for example, accuracy or similarity judgments at different time points. Furthermore, as we only observed effects between ∼100 msec and ∼200 msec post stimulus, we cannot exclude that the strong signal-to-noise ratio in this time-window influences the result. Using a more precise neural measurement could reveal whether other time-points are also involved in decision-making.

### Summary

In this work, we explored the link between neural representations and behavior using empirical and computational methods. Empirically, we showed that brain and behavior are linked during peak brain decoding, that is, when neural representations of different scenes are most distinguishable. Computationally, we demonstrated that a recurrent CNN can serve as a unified model of scene processing and predicts scene categorization in humans better than a feedforward model, suggesting that future studies can use RCNNs to further understand scene processing in humans in terms of both neural and behavioral data.

## Acknowledgments

We thank the HPC Service of ZEDAT, Freie Universität Berlin (Bennett et al., 2020), for computing time, as well as NVIDIA Corporation for the donation of the GPU used for this research. We gratefully acknowledge the contribution of Alessandro Gifford, Greta Häberle, Johannes Singer, and Siying Xie and their valuable comments on the manuscript. We would also like to thank Martin Hebart, Youssef Kashef, Varun Kapoor, Thomas Carlson, J. Brendan Ritchie, and Tijl Grootswagers for their input on the analysis.

Reprint requests should be sent to Agnessa Karapetian, Freie Universität Berlin, Habelschwerdter Allee 45, Room JK 25/229, 14195 Berlin, Germany, or via e-mail: agnek95@zedat.fu-berlin.de.

## Data Availability Statement

The code used for this project can be found under https://github.com/Agnessa14/Perceptual-decision-making. The data and stimulus set can be found on https://osf.io/4fdky/.

## Author Contributions

Agnessa Karapetian: Conceptualization; Data curation; Formal analysis; Investigation; Methodology; Visualization; Writing—Original draft; Writing—Review & editing. Antoniya Boyanova: Data curation; Validation; Writing—Review & editing. Muthukumar Pandaram: Formal analysis; Methodology; Visualization. Klaus Obermayer: Conceptualization; Methodology; Resources; Supervision; Validation; Writing—Review & editing. Tim C. Kietzmann: Conceptualization; Methodology; Resources; Supervision; Validation; Writing—Review & editing. Radoslaw M. Cichy: Conceptualization; Funding acquisition; Investigation; Methodology; Project administration; Resources; Supervision; Validation; Writing—Review & editing.

## Funding Information

A. K. is supported by a PhD fellowship of the Einstein Center for Neurosciences. R. M. C. is supported by the German Research Council (https://dx.doi.org/10.13039/501100001659), grant numbers: CI 241/1–1, CI 241/3–1, CI 241/1–7; and the European Research Council (https://dx.doi.org/10.13039/501100000781), grant numbers: ERC-StG-2018-803370. T. M. C. is supported by the European Research Council, grant number: ERC-StG-2022-101039524.

## Diversity in Citation Practices

Retrospective analysis of the citations in every article published in this journal from 2010 to 2021 reveals a persistent pattern of gender imbalance: Although the proportions of authorship teams (categorized by estimated gender identification of first author/last author) publishing in the *Journal of Cognitive Neuroscience* (*JoCN*) during this period were M(an)/M = .407, W(oman)/M = .32, M/W = .115, and W/W = .159, the comparable proportions for the articles that these authorship teams cited were M/M = .549, W/M = .257, M/W = .109, and W/W = .085 (Postle and Fulvio, *JoCN*, 34:1, pp. 1–3). Consequently, *JoCN* encourages all authors to consider gender balance explicitly when selecting which articles to cite and gives them the opportunity to report their article's gender citation balance. The authors of this article report its proportions of citations by gender category to be as follows: M/M = .662; W/M = .203; M/W = .081; W/W = .054.
